# Development of Polymer Composite Membrane Electrolytes in Alkaline Zn/MnO_2_, Al/MnO_2_, Zinc/Air, and Al/Air Electrochemical Cells

**DOI:** 10.3390/polym16213068

**Published:** 2024-10-31

**Authors:** Sheng-Jen Lin, Juin-Yih Su, Dave W. Chen, Gwomei Wu

**Affiliations:** Institute of Electro-Optical Engineering, Chang Gung University, Chang Gung Memorial Hospital, Taoyuan 333, Taiwan; aa691010@yahoo.com.tw (S.-J.L.); su1491@adm.cgmh.org.tw (J.-Y.S.); mr5181@cgmh.org.tw (D.W.C.)

**Keywords:** polymer composite membrane, Zn/MnO_2_, Al/MnO_2_, power density, discharge capacity, electrochemical stability

## Abstract

This paper reports on the novel composite membrane electrolytes used in Zn/MnO_2_, Al/MnO_2_, Al/air, and zinc/air electrochemical devices. The composite membranes were made using poly(vinyl alcohol), poly(acrylic acid), and a sulfonated polypropylene/polyethylene separator to enhance the electrochemical characteristics and dimensional stability of the solid electrolyte membranes. The ionic conductivity was improved significantly by the amount of acrylic acid incorporated into the polymer systems. In general, the ionic conductivity was also enhanced gradually as the testing temperature increased from 20 to 80 °C. Porous zinc gel electrodes and pure aluminum plates were used as the anodes, while porous carbon air electrodes or porous MnO_2_ electrodes were used as the cathodes. The cyclic voltammetry properties and electrochemical impedance characteristics were investigated to evaluate the cell behavior and electrochemical properties of these prototype cells. The results showed that these prototype cells had a low bulk resistance, a high cell power density, and a unique device stability. The Al/MnO_2_ cell achieved a density of 110 mW cm^−2^ at the designated current density for the discharge tests, while the other cells also exhibited good values in the range of 70–100 mW cm^−2^. Furthermore, the Zn/air cell consisting of the PVA/PAA = 10:5 composite membrane revealed an excellent discharge capacity of 1507 mAh. This represented a very high anode utilization of 95.7% at the C/10 rate.

## 1. Introduction

Owing to the limited supply of natural energy reserves and the ever-rising concerns for our global ecological safety, the demand for more efficient and stable energy storage and consumption technologies has been increasing rapidly over the years [1,2,3]. For energy storage applications, electrochemical cells are undeniably important devices. They have received much attention due to their combination of characteristics, including good energy density, lower cost, and stability for a longer lifespan [4,5,6,7]. In addition, the Zn/MnO_2_ cell offers many benefits, including a low cost, natural abundance, high safety, extensive electrochemical constancy window, mechanical flexibility, low toxicity, and thermal stability [8,9]. It can be utilized in continuous or intermittent applications, and therefore holds a unique position in the portable battery industry. The theoretical capacity of these cells can be achieved by reducing Mn^4+^ to Mn^2+^ while accommodating MnO_2_ with the insertion of Zn^2+^ in order to reach 616 mA h g^−1^. In addition, the various crystal structural forms of the MnO_2_ can affect the electrochemical performance. The corresponding microstructure and morphology inevitably play an important role in the recombination of cathodes and, therefore, the Zn cell storage results. Nevertheless, their less attractive declining discharge curves may limit their use in some devices. On the other hand, the zinc/air cell extracts oxygen straight from the ambient air to generate energy for chemical reactions. This cell is therefore sometimes called the semi-fuel cell. Many researchers have focused on the Zn/air cell because of its significant energy density, safety, and uniform discharge voltage [10,11,12]. It has been demonstrated that a unique redox mediation design would liberate the oxidation–reduction reactions from the restricted electrode compartments [13]. This, in turn, could provide a possible means of replenishing the zinc metal fuel in the electrochemical cell system. Therefore, this design could enable cell operations to become a lot more flexible. The system can eventually be scaled up by using a modular design to meet the stringent demand for energy. Furthermore, a recent study proposed a hybrid power generator with an external zinc storage system using the redox mediation design [14]. The researchers combined the flexibility of the anode side and the high power and durability of the air electrode part. They achieved a highly improved peak cell power density of 0.51 W cm^−2^, and also showed an impressive discharge capacity higher than 48 A-h. This strategy significantly diminished the self-discharge problem in the zinc electrode in the Zn/air cells. The scientific community has demonstrated better electrochemical performances and opened a gateway for future battery technology. Meanwhile, aluminum metal electrodes have long attracted attention as a potential battery anode material due to aluminum’s natural abundance and high theoretical A-h capacity, voltage, and specific energy. It is, therefore, interesting to also assemble and compare a simple Al/MnO_2_ cell and Al/air cell in order to explore the potential application of such electrochemical devices.

The solid polymer electrolyte (SPE) membrane is considered to be of paramount importance in the electrochemical battery system [15,16,17,18,19]. It engages liquid electrolytes and avoids harmful contact between the cathode and anode electrodes, which would cause a battery to short circuit. However, the larger pore size of a non-woven-type membrane separator cannot completely prevent the redox reaction created by dendrite metal from entering the other side, which can trigger harmful explosions in a battery system. Over the past decade, rigorous safety assurance and improvements to the cycling life have become inevitable, largely caused by the rapid progress witnessed in portable consumer electronics and electric vehicle applications [18]. In addition, lower and unstable electrolyte absorption would cause cell electrolyte leakage and electrolyte drying. All these issues could limit the practical application of batteries. While multi-stacks of non-woven membrane separators might somewhat resolve this problem, thicker stacks of separators possess less space for the active electrode materials. This will, in turn, significantly decrease the cell’s electrochemical performance. Thus, using a new composite polymer electrolyte membrane to replace the traditional polypropylene/polyethylene (PP/PE) separator is a significant challenge, and is of interest for energy storage systems.

Solid polymer electrolytes have become widely accepted as a useful technique to resolve safety issues, especially in high-energy-density batteries [20,21]. Fenton et al. reported the first theoretical analysis of solid polymer electrolytes and devoted their study to conducting characteristics of an ionic nature [22]. They discovered the dissolution of alkali metal salts in polyethylene oxide that could form a conductive complex. Later, Armand et al. discussed the potential application of solid-type polymer electrolytes in lithium-based batteries, showing an ionic conductivity near 10^−4^ S cm^−1^ [23]. In addition, Fauvarque et al. demonstrated that their polyethylene oxide–KOH polymer electrolytes had an ionic conductivity in the order of 10^−3^ S cm^−1^ [24,25]. The addition of non-organic fillers could also enhance the ionic conductivity of the polyethylene oxide polymer electrolyte system. The attained composite electrolytes generally exhibited an ionic conductivity that was ten times higher compared to the purely PEO polymer electrolyte [16,19]. This was generally attributed to the increase in amorphous phase, which has a higher ionic mobility. A more commercially positive improvement in the sulfonated system was Nafion^®^. This material system has been utilized as a separator in many electrochemical devices [26,27]. It has achieved a conductivity ranging from 0.01 to 0.1 S cm^−1^. However, the high cost of manufacturing Nafion could still limit its widespread application in energy storage electrochemical systems [28]. Merle et al. studied poly(vinyl alcohol) (PVA) by performing a cross-linking reaction and presented good experimental results, at 72 mW cm^−2^ [29]. In order to further increase the safety, reliability, and processibility of many battery applications, it has become necessary to develop more suitable materials to advance this solid polymer electrolyte technology [30].

In this paper, polymer composite electrolyte membranes are presented using sulfonated PP/PE (s-PP/PE) non-woven separators and highly conductive polymeric mixtures. The composite electrolyte membranes can provide a high mechanical strength and also an improved ionic conductivity. They are also versatile during battery manufacture procedures of all kinds and sizes due to their high plasticity. Polyvinyl alcohol and polyacrylic acid (PAA) resins are among the best polymeric candidates due to their excellent hydrophilic properties and their ability to form films in aqueous solution systems. PP/PE non-woven separator sheets can be used as composite reinforcements to improve mechanical properties. It is necessary to perform a sulfonation chemical treatment to improve the hydrophilic properties that enable the formation of a well-bonded composite membrane structure with the PVA/PAA polymeric blend. Simple Al/MnO_2_, Zn/MnO_2_, Al/air, and zinc/air cells utilizing s-PP/PE/PVA/PAA solid polymer electrolyte membranes that contain KOH solution were assembled for evaluation. The cyclic voltammetry spectra and AC impedance techniques were all applied to investigate the battery device characteristics and the electrochemical properties of these prototype cells.

## 2. Materials and Methods

In the experiments, non-woven polypropylene/polyethylene separator sheets were employed to prepare composite membrane electrolytes for the electrochemical cells. These were used in order to improve the dimensional stability as well as the electrochemical properties. The starting separator sheets were supplied by Coin Nanotech (Taoyuan, Taiwan). The nominal sheet thickness was approximately 0.2 mm, and they were designed to have a porosity of 65–70%. Their area weight was approximately 60 g m^−2^. The separator material was rinsed in de-ionized water, with subsequent drying in a convention oven; it was then immersed in a 98 wt% H_2_SO_4_ solution at 90 °C. After the so-called sulfonation chemical reaction, suitable functional groups were created. The sulfonated separators were then cleansed constantly in fresh de-ionized water under shaking. The separator sheet samples were thus obtained via further drying.

In the second stage, polyvinyl alcohol (M.W. 75k, Chang Chung Chemicals, Taipei, Taiwan), acrylic acid (AA) monomer (Aldrich, St. Louis, MO, USA), and KOH (Merck, Darmstadt, Germany) were purchased to further establish the composite membrane electrolytes. Appropriate fractions of AA monomer and cross-linking agent (triallylamine, Aldrich, St. Louis, MO, USA) were added and blended in pure H_2_O under stirring at 60 °C for 12 h. They were varied at 10:3, 10:5, and 10:7.5 by weight with respect to PVA. These prepared solutions were also combined with 75 mol% potassium hydroxide. The combination was further integrated into the polymer solution, and then the s-PP/PE non-woven sheets were impregnated. In addition, an appropriate amount (10% by weight percent, with respect to AA composition) of (NH_4_)_2_S_2_O_8_ (Aldrich, St. Louis, MO, USA) was used as an initiator. This was eventually included in the above sticky polymer solution for free-radical polymerization. The composites of the s-PP/PE membrane sheets that had been impregnated with the sticky homogeneous mixture were dispensed on a PTFE platter. In a fumehood, the unnecessary water content was eventually removed by the air flow. Finally, the self-supporting composite polymer electrolyte membranes were obtained.

To absorb more conducting ions, the s-PP/PE/PVA/PAA samples were submerged in a 32 wt% potassium hydroxide solution for 24 h. After this step, all the samples were weighed again. The sample thickness was measured to be approximately 0.4–0.5 mm. The electrical conductivity evaluations were conducted using the impedance method. The s-PP/PE/PVA/PAA composite membranes were fixed between the stainless steel electrodes in a spring-loaded glass holder. The contact surface area was approximately 0.785 cm^2^. The AC impedance study was conducted by utilizing the AutoLab system (Artisan Technology Group, Champaign, IL, USA). The frequency range was studied from 100 to 100,000 Hz. The experimental temperature range evaluated was 25 °C to 80 °C. The cyclic voltammetric curves were also investigated using the same system. For this study, the composite electrolyte membrane was inserted between a counter electrode and a working electrode, for a symmetrical Zn|s-PP/PE/PVA/PAA|Zn or the Al system.

During the preparation of the Zn/air batteries, a carbon slurry was made for the gas diffusion layer [31]. On the other hand, the active air electrode was fabricated by showering a blend of poly(tetrafluoroethylene) and KMnO_4_ with IPA [10]. Finally, the electrodes were assembled and sintered at 360 °C under 800 N cm^−2^ for 30 min. The Zn gel was assembled as an anode in Zn/MnO_2_ and Zn/air cells. The MnO_2_ electrode was also prepared as a cathode in the Zn/MnO_2_ and Al/MnO_2_ cells by mixing 96 wt% electrolytic manganese dioxide (EMD), 3 wt% acetylene black, and a 1 wt% PTFE binder solution. It was then placed onto a porous nickel foam substrate and pressed at 1000 N cm^−2^. The Al anode electrode was prepared using high-purity aluminum (99.99%) and finely polished, utilizing different grain-sized sandpapers. Then, the surface grease was cleaned using acetone and distilled water. The experimental cells of solid-state Zn/MnO_2_, Zn/air, Al/MnO_2_, and Al/air with the alkaline s-PP/PE/PVA/PAA composite electrolytes were assembled in a 2 × 3 cm^2^ rectangular can that was equipped with stainless steel current collector. The samples prepared from the various s-PP/PE/PVA/PAA electrolytes were then discharged at the various rates outlined in this study. The bulk resistances (R_b_) were measured both before and after the discharge process. The battery characteristics were investigated using the BAT 778 model (AcuTech Systems, New Taipei, Taiwan) charge/discharge unit.

## 3. Results and Discussion

### 3.1. Morphology and Mechanical Properties

**Figure 1:**
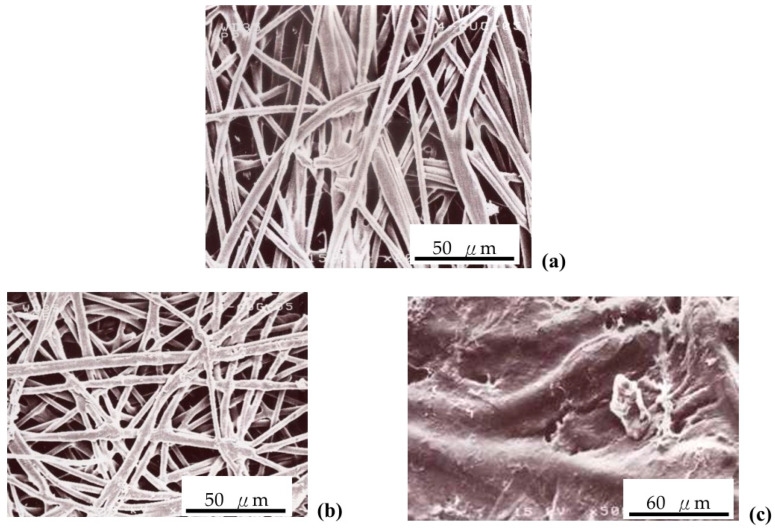
SEM micrographs of (**a**) the un-sulfonated and (**b**) sulfonated PP/PE membrane, and (**c**) the top surface of s-PP/PE/PVA/PAA.

**Figure 2:**
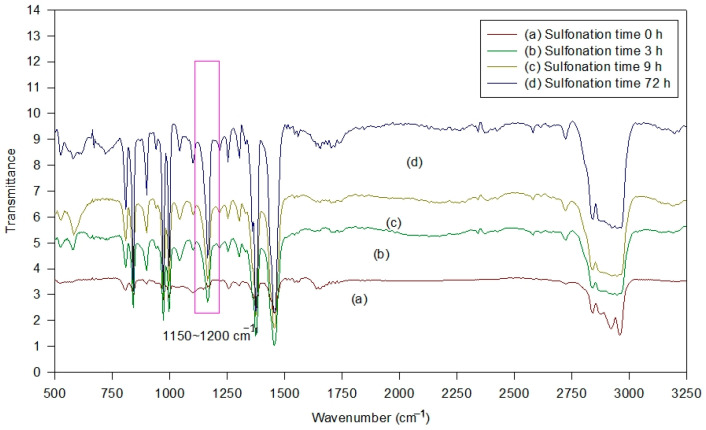
IR spectra of the membrane samples.

Figure 1 shows the SEM micrographs of the PP/PE separator without sulfonation, and also the sulfonated s-PP/PE/PVA/PAA composite polymer membrane after the 72 h sulfonation treatment. The PVA/PAA ratio was 10:5. This non-woven PP/PE separator sheet exhibited many filaments with a diameter of 10–20 μm. The sulfonated s-PP/PE filaments were impregnated and bound with the PVA/PAA blend. The top surface of the membrane was uniform, which is important in any battery system. In addition, Figure 2 displays the infrared (IR) spectra of the membrane samples. The additional highlighted transmittance peaks belong to asymmetric SO_3_^−^, indicating that suitable functional groups were created on the PP/PE separator after the sulfonation step.

**Figure 3:**
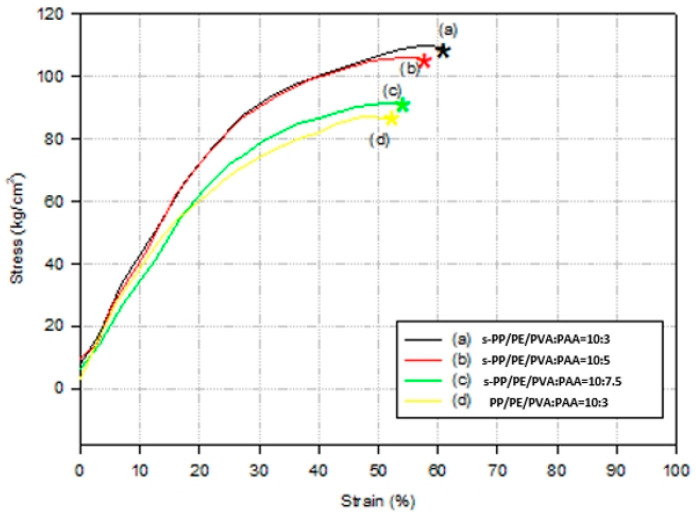
Mechanical testing curves for the membrane specimens.

The room-temperature tensile mechanical testing curves for the membrane specimens are shown in Figure 3. The sulfonated membrane samples were not only stiffer, but also stronger with regard to their tensile strength. They also showed a slightly higher elongation at break, which ensured that there was interplay between PVA and PAA with the sulfonated PP/PE separators. In addition, mechanical integrity is also beneficial to membrane stability. However, the higher acrylic acid content seemed to increase the ductility and affected its swelling.

### 3.2. Swelling and Absorption

The s-PP/PE/PVA/PAA solid polymer membranes exhibited a swelling behavior in the KOH alkaline solution. Table 1 shows the swelling and the absorption ratio data for the s-PP/PE/PVA/PAA composite membranes in a 6 M KOH aqueous solution. The immersion time was controlled at 24 h. The swelling ratio slightly increased when the PAA content was also increased. It was experimentally shown that the absorption ratio of the composite membrane samples would further increase by about 40% as the PVA/PAA blend ratio increased. This is shown by the significant increase in hydrophilic groups in the membrane matrix. The PVA/PAA ratio also improved the ionic transport, as evidenced by the analysis of the AC impedance spectra. Nevertheless, this high absorption ratio could be very helpful for polymer electrolytes, because it can provide more aqueous media for ionic transport in a battery system.

### 3.3. Ionic Conductivity

**Figure 4:**
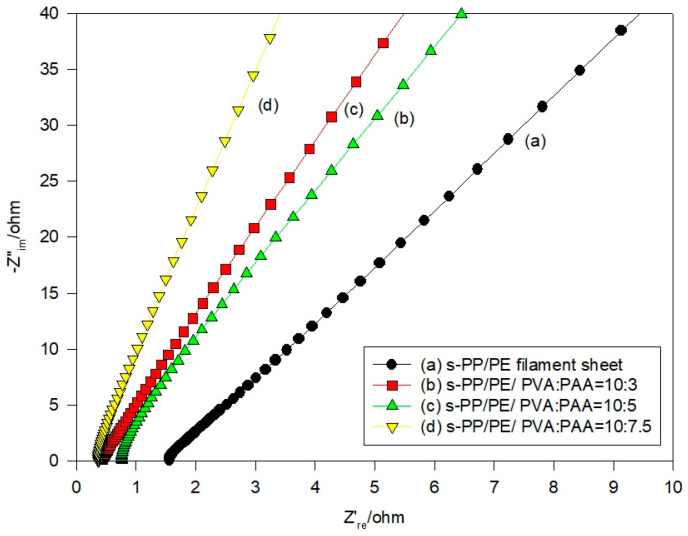
Nyquist plots for the polymer composite samples at room temperature.

The Nyquist plots for the polymer composite electrolyte samples are shown in Figure 4. An examination of these spectra provided information about the resistance of the s-PP/PE/PVA/PAA samples. They were derived from the higher frequency region. The R_b_ data (in Ω) were regarded as the ionic conductivity [10]. Table 2 summarizes the ionic conductivity data for the various composite electrolyte samples at the corresponding temperatures. It was observed that the ionic conductivity increased as the composition of the acrylic acid increased at all of the experimental temperatures. This is in good agreement with the KOH solution absorption ratio. Alternatively, it was observed that there was a higher ionic conductivity at a higher atmospheric temperature. This may benefit the composite membrane electrolytes used in the battery system and enable a good cell performance.

### 3.4. Cyclic Voltammetry Analysis

**Figure 5:**
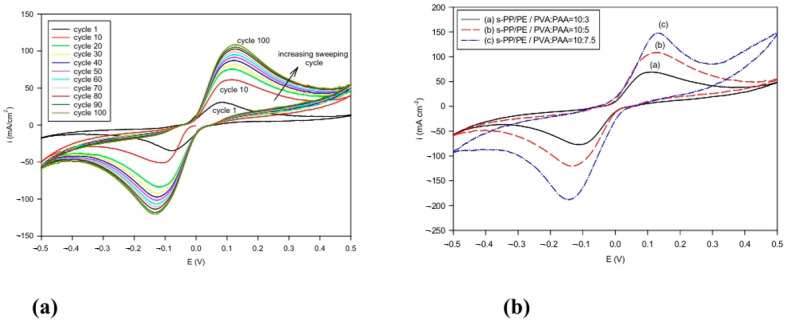
Cyclic voltammetry spectra for alkaline s-PP/PE/PVA/PAA composite membrane using PVA/PAA = 10:5 in Zn|membrane|Zn cell at 25 °C. (**a**) Different sweeping cycles, and (**b**) different PVA/PAA compositions at the 100th sweeping cycle.

Cyclic voltammetry is usually employed to examine the electrochemical stability of composite electrolytes. Figure 5a shows the spectra from the sulfonated electrolyte sample using a PVA/PAA = 10:5 composition. The composite electrolytes were swept between –0.50 V and +0.50 V with Zn|electrolyte|Zn. The scan rate was 10 mV s^−1^. Zn was used for both electrodes in order to focus on evaluating the stability of the polymer composite electrolytes, so that we would not be concerned with the influence of a different metallic electrode. After 100 sweeping cycles, the cathodic and the anodic peaks remained symmetric in the range of −0.12 V to +0.12 V and had high stability in the zinc electrode system. None of the sweeping curves shook, but instead were smooth. In addition, both the oxidation current density and the reduction current density increased as the number of cycles increased. This was generally caused by the initial activation of Zn/ZnO during the oxidation and reduction process. This trend was observed up to 100 cycles in the experiment, suggesting that Zn/ZnO could be employed in Zn/air cells. Figure 5b shows the different blend ratio samples, all at the 100th sweeping cycle. Again, for the higher PAA content samples, a greater reduction and oxidation current could be observed. Nevertheless, symmetric anodic and cathodic peaks could also be attained.

**Figure 6:**
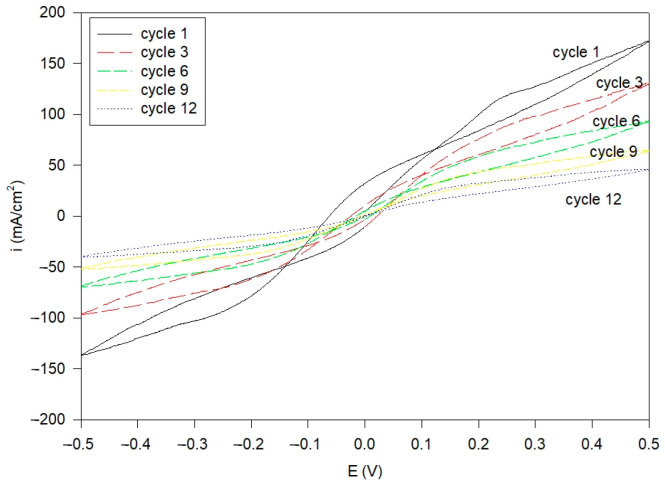
Cyclic voltammetry for the s-PP/PE/PVA/PAA composite membranes in the Al|membrane|Al cell at 25 °C with different sweeping cycles.

Figure 6 shows the cyclic voltammograms for the composite electrolytes using a PVA/PAA polymer blend composition of 10:5 in the Al electrolyte cells. The cyclic sweeping ranged between –0.5 V and +0.5 V. The level in the oxidation and reduction current reduced as the cycles increased from the first cycle to the twelfth cycle, and the maximum current density was significantly reduced from 100 to 10 mA cm^−2^ in the range of E_cathodic_ = −0.2 V to E_anodic_ = +0.2 V. This is attributed to the inevitable formation of the deactivation layer in the alkaline system. During this initial stage, only up to 12 cycles, the deactivation layer of the electrode became thick when the cycles increased. Thus, the cycle life of the Al/air cell was not as good as that of the Zn/air system. Nevertheless, it had a reasonable current density during oxidation and reduction and a symmetrical curve.

### 3.5. Electrochemical Performance of Solid Cells

**Figure 7:**
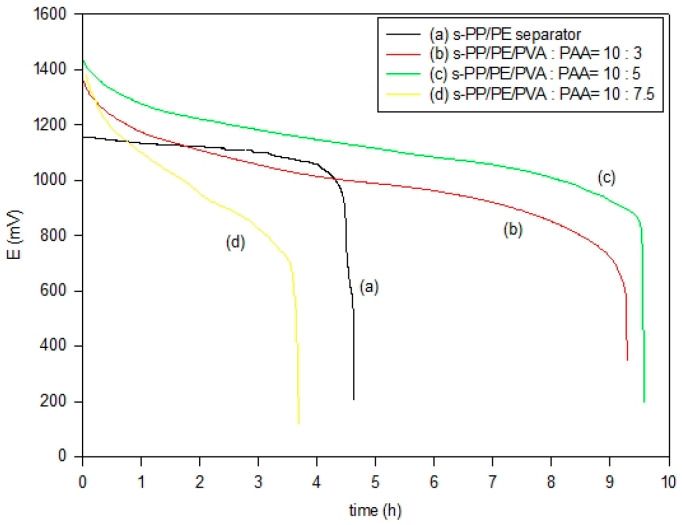
Discharge curves for Zn/air cells using different s-PP/PE/PVA/PAA composite membrane electrolytes at a C/10 rate.

Figure 7 presents the discharge curves of the Zn/air cells utilizing s-PP/PE/PVA/PAA alkaline composite membrane electrolytes. These tests were conducted by considering the C/10 rate under ambient temperature. The corresponding electrochemical results are shown in Table 3. It is worth mentioning that the data were taken from the first discharge cycle. The Zn/air cell using the s-PP/PE separating membrane had a capacity of 728 mAh, which exhibited inferior Zn utilization. This result might indicate anode expansion and dendrite production, thus causing cell failure. On the other hand, the Zn/air cell utilizing an s-PP/PE/PVA/PAA composite electrolyte at PVA/PAA = 10:5 had the best discharge capacity, at 1507 mAh. The best anode utilization was thus 95.7%. It seems that the composite membrane electrolyte had a unique morphology that could successfully prevent the penetration of zincate, as well extend the cell discharge life. It must also be mentioned that these metal–air cells are semi-open. During the long-term discharge experiments, the cell’s electrolyte could have easily evaporated. Nevertheless, this would have directly influenced the high electrolyte absorption ratio for the highest PVA/PAA ratio of 10:7.5. Thus, the results showed the lowest capacity at 581 mAh, and also the lowest anode utilization rate at 37.0%. After the cell discharge tests, we disassembled this cell and discovered that this composite membrane with the highest PAA content should have absorbed more electrolytes and become curled, therefore decreasing the cell discharge life.

**Figure 8:**
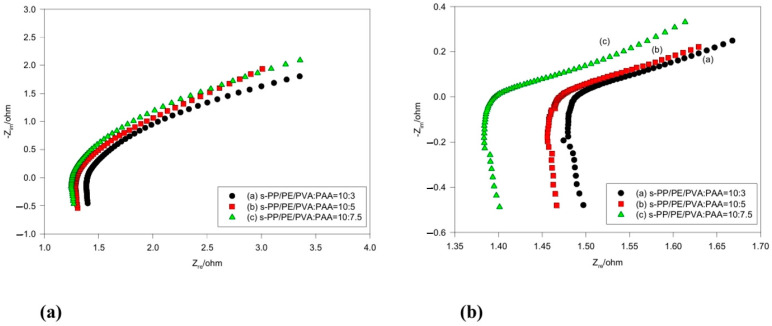
AC impedance spectra for the alkaline Zn/air cells at 25 °C (**a**) before and (**b**) after the C/10 discharge tests.

It has been shown that the bulk resistance of the cell utilizing the various membrane electrolytes was in the range of 1.27–1.39 ohm, as shown in Figure 8a. The results were measured via AC impedance spectroscopy before the discharge tests. However, Figure 8b shows that, after the discharge tests, the bulk resistance of the solid-state cells was only moderately elevated, now ranging between 1.40 and 1.49 ohms. It is clear that the shape of the plot is not a semicircle, and that the AC impedance spectra clearly contain two parts. It appears that the higher frequency part is more likely influenced by the ionic conduction process inside the composite membrane. However, the lower frequency part is somewhat different. Thus, the resistance could be caused by a more complex phenomenon, such as the diffusion of the transport ions and non-homogeneity at the electrolyte/electrode interface [31].

**Figure 9:**
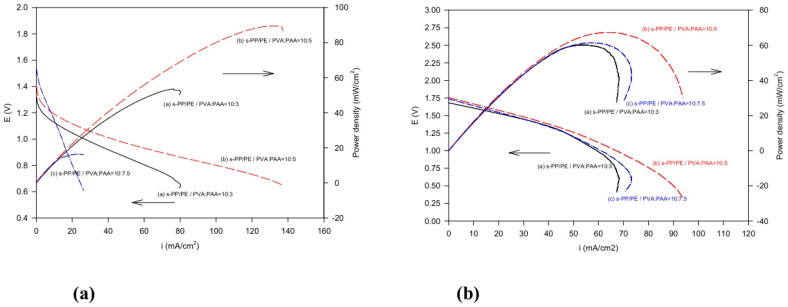
Current–potential and power–current curves for the (**a**) Zn/air cells and (**b**) Al/air cells.

Figure 9a,b separately illustrate the cell performance of the Zn/air cells and Al/air cells, alongside the discharge current density. The cell power density results are also provided. It is clear that the PVA/PAA = 10:5 composite electrolyte Zn/air cell showed the best power density value at 91 mW cm^−2^. This performance is much better than that when only using the PVA/PAA electrolyte and a polymer-gelled Zn/air cell [32]. This can be attributed to the excellently bound structures in the sulfonated PP/PE sheet fibers and the appropriate PVA/PAA polymer blends that provide dimensionally stable composite membranes in the battery systems. The cells can thus sustain a higher discharge current density without becoming broken, deformed, or curled.

**Figure 10:**
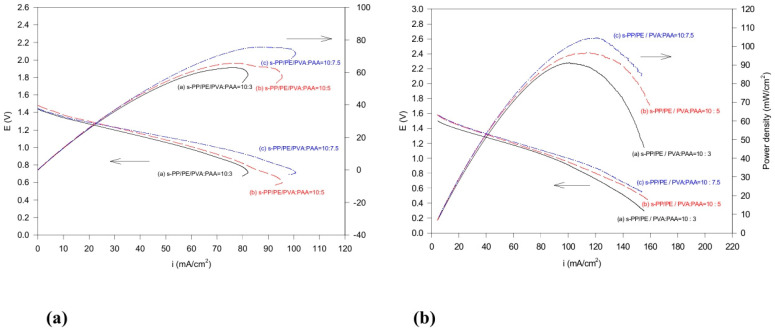
Current–potential and power–current curves for (**a**) Zn/MnO_2_ cells and (**b**) Al/MnO_2_ cells.

The performance of the cells, as a function of the discharge current density of the Zn/MnO_2_ and Al/MnO_2_ cells assembled with the sulfonated composite membrane electrolytes and the various polymer blend ratios, is shown in Figure 10a and Figure 10b, respectively. Unlike the metal–air cells, which are semi-open battery systems, the Zn/MnO_2_ and Al/MnO_2_ cells are closed battery systems. There are no problems related to water evaporation if the cell can be absolutely sealed. The power density results are increased as the acrylic acid composition increases. This is attributed to the higher ionic conductivity of the sulfonated composite membranes due to greater electrolyte absorption. The Al/MnO_2_ cell with a 10:7.5 blend ratio exhibits the best power density at 110 mW cm^−2^, while the discharge current density is 120 mA cm^−2^. This is superior to the Zn/MnO_2_ cell (76 mW cm^−2^ at ~90 mA cm^−2^). This advantage might be credited to the high theoretical A-h capacity of the aluminum electrode, as well as its high cell voltage (2.6 V vs. 2.1 V) and high specific energy.

It can be concluded that the recommended polymer blend ratio is 10:5 for the semi-open metal–air cell system (i.e., Zn/air and Al/air in this report), and that it becomes 10:7.5 for the absolutely closed system (Zn/MnO_2_ and Al/MnO_2_). The experimental results clearly evidence that the Zn/air cells would maintain satisfactory oxidation and reduction current densities after a much higher number of sweeping cycles. Their symmetrical anodic and cathodic peaks could also be achieved. The solid-state Zn/air cell revealed the highest discharge capacity at 1507 mAh and the best anode utilization percentage of 95.7%. On the other hand, the Al electrode system lost much of its current density with only a dozen sweeping cycles. It is thus not as suitable for application in semi-open cell systems. However, the Al/MnO_2_ cell exhibits the best power density at 110 mW cm^−2^, making it the best option for absolutely closed semi-open cell systems.

The present study focuses on laboratory-scale cells. We suggest that the scalability of the synthesis process and its potential for real-world applications are investigated further. On the other hand, it would be interesting to discuss the environmental and economic impacts of the developed materials. In the next step, we would be glad to provide a comprehensive analysis of the sustainability of these materials in comparison to the other electrolyte technologies available. The aim of this research is to address the potential challenges associated with scaling up these materials for commercial use in the near future.

## 4. Conclusions

In this report, we have developed and evaluated a group of alkaline composite membrane electrolytes for electrochemical cells. These electrolytes offer advantageous properties, with a high ionic conductivity and highly dimensionally stable structures. The optimal design for the sulfonated composite membrane electrolyte comprised an appropriate sulfonation treatment for the PP/PE separator sheet and a suitable ratio of polyvinyl alcohol to polyacrylic acid. The recommended blend ratio was 10:5 for the semi-open cell system and 10:7.5 for the absolutely closed system. This composite membrane exhibited a high ionic conductivity of 0.157 S cm^−1^ at 25 °C and had good mechanical stability. The micrographs of the composite membrane exhibited excellent microstructural integrity in the sulfonated fiber and polymer blends. The possible applications of these electrolytes have been demonstrated by the prototype solid-state alkaline batteries fabricated using the Zn/air, Zn/MnO_2_, Al/air and Al/MnO_2_ cells. The experimental results of this study showed that the Al/MnO_2_ cell had the best power density at 110 mW cm^−2^, while the other cells also achieved a power density of approximately 70–100 mW cm^−2^. Sulfonated PP/PE/PVA/PAA alkaline polymer composite membrane electrolytes can thus be further developed for potential application in many battery systems and electrochemical devices.

## Figures and Tables

**Table 1:** Swelling ratio and absorption ratio properties of the composite membranes at room temperature in a 6 M KOH solution. The immersion time was 24 h.

Property	s-PP/PE/PVA–PAA (10:3)	s-PP/PE/PVA–PAA (10:5)	s-PP/PE/PVA–PAA (10:7.5)
Swelling ratio (%)	52.6	58.1	61.3
Absorption ratio (%)	110.9	138.1	154.7

**Table 2:** Ionic conductivity (S cm^−1^) of the electrolyte samples at the various temperatures.

Temperature (°C)	s-PP/PE/PVA–PAA (10:3)	s-PP/PE/PVA–PAA (10:5)	s-PP/PE/PVA–PAA (10:7.5)
25	0.090	0.157	0.210
40	0.093	0.166	0.232
50	0.096	0.177	0.246
60	0.100	0.183	0.265
70	0.101	0.194	0.279
80	0.109	0.202	0.291

**Table 3:** Experimental cell results for Zn/air utilizing the s-PP/PE/PVA/PAA composite membrane electrolytes at a C/10 rate.

Property	s-PP/PE/PVA–PAA (10:3)	s-PP/PE/PVA–PAA (10:5)	s-PP/PE/PVA–PAA (10:7.5)
Design capacity (mAh)	1574	1574	1574
Discharge current (mA)	150	150	150
Discharge time (h)	9.3	9.5	3.6
Real capacity (mAh)	1465	1507	581
Utilization (%)	93.1	95.7	37.0
Cell bulk resistance before discharge (Ω)	1.39	1.31	1.27
Cell bulk resistance after discharge (Ω)	1.49	1.47	1.40

## Data Availability

The original contributions presented in this study are included in the article and further inquiries can be directed to the corresponding author.
